# The data are inadequate to assess safety and efficacy of mass chemotherapy for *Taenia solium* taeniasis

**DOI:** 10.1371/journal.pntd.0008294

**Published:** 2020-07-16

**Authors:** A. Clinton White, Seth O’Neal, Andrea Winkler, Annette Abraham, Hélène Carabin

**Affiliations:** 1 Infectious Disease Division, Department of Internal Medicine, University of Texas Medical Branch, Galveston, Texas, United States of America; 2 School of Public Health, Oregon Health & Sciences University and Portland State University, Portland, Oregon, United States of America; 3 Department of Neurology, Center for Global Health, Technical University of Munich, Germany; 4 Centre for Global Health, Institute of Health and Society, University of Oslo, Norway; 5 Département de Pathologie et Microbiologie, Faculté de médecine vétérinaire, Département de médecine sociale et préventive, École de santé publique, Centre de Recherche en Sant/ Publique, Université de Montréal, Canada; Istituto Superiore Di Sanita, ITALY

## Abstract

As members of the Pan American Health Organization (PAHO) and World Health Organization (WHO) guidelines development group on chemotherapy strategies for the control of *Taenia solium* taeniasis, we are very disappointed at the systematic review by Haby and colleagues. With respect to the analysis of efficacy, the authors did not account for differences in the methods used to ascertain the outcome in the studies analyzed. There are also major concerns regarding the safety analyses. Few of the included studies used carefully designed active surveillance protocols to detect epileptic seizures and/or chronic progressive headaches. These neurologic side effects, due the inadvertent killing of viable brain cysts, have been noted after mass therapy with praziquantel and albendazole. We wholeheartedly agree with the authors’ statement in their discussion that control programs applying chemotherapy using mass drug administration “need to be informed by evidence of the best drug and dose in terms of efficacy and side-effects.” Unfortunately, the flawed analysis that was published is contrary to that goal.

As members of the Pan American Health Organization (PAHO) and World Health Organization (WHO) guidelines development group on chemotherapy strategies for the control of *Taenia solium* taeniasis, we are very disappointed at the systematic review by Haby and colleagues. [1] In prior deliberations and discussions with the authors, we highlighted major methodologic flaws in the current analysis. However, these flaws were not addressed in this meta-analysis, and the manuscript was published without us seeing it beforehand. Unfortunately, the published version includes substantial biases that, if considered properly, would have likely contradicted the current conclusions. In particular, we disagree with the authors’ conclusions in support of the efficacy and safety of albendazole (ABZ) and praziquantel in mass drug administration.

With respect to the analysis of efficacy, the authors did not account for differences in the methods used to ascertain the outcome in the studies analyzed. Stool-based diagnosis of taeniasis can be accomplished through visualization of eggs using microscopy, detection of coproantigens (coAg) via immunoassays, and amplification of DNA using polymerase chain reaction. A critical oversight in this meta-analysis was disregarding the disparity in the sensitivity and specificity of these methods. All of the studies involving ABZ included in the meta-analysis relied solely on microscopy to assess treatment efficacy, [2, 3] a method that is notoriously insensitive and cannot distinguish *T*. *solium* from *T*. *saginata*. Microscopy is also subject to inaccurate interpretation of the temporary cessation of egg shedding that may occur when terminal proglottids are killed but the scolex remains viable. Both limitations would result in misclassification of persistent infections as cured and would therefore be expected to overestimate the efficacy of ABZ. In contrast, coAg detection was disproportionately used in studies of niclosamide and praziquantel. coAg detection is much more sensitive than microscopy and remains positive during temporary cessation of egg shedding. [4] In some of the included studies, the outcome was assessed through coAg detection of serial stool samples, further increasing the sensitivity. These latter approaches all limit the likelihood of misclassification of persistent infections as cured. Direct comparison of studies that measure treatment efficacy with microscopy versus coAg detection, as was done in this meta-analysis, will invariably bias in favor of studies that use the method with lower sensitivity. The fact that most of these tests also have different specificity values cannot be discounted as it will invariably introduce various levels of biases across studies. Moreover, the authors pooled the results (see their Fig 3 and Fig 4) from all studies, which used different drugs, doses, treatment regimens, and diagnostic tests, increasing the risk for flawed conclusions.

There are also major concerns regarding the safety analyses. Few of the included studies used carefully designed active surveillance protocols to detect epileptic seizures and/or chronic progressive headaches, the main neurologic side effects that would be anticipated in the inadvertent killing of viable brain cysts. Epileptic seizures are highly stigmatizing in most countries where cysticercosis is endemic, which would lead to underreporting of these symptoms, especially in studies not specifically designed to measure these effects. In addition, none of the studies followed participants during the period in which neurologic side effects would be anticipated to occur (days 3 to 5 after treatment). Thus, we do not feel that there is any good data to support safety of ABZ or praziquantel in areas that are endemic for *T*. *solium*. In fact, results from a prospective study on neurologic side effects after mass drug administration of praziquantel for schistosomiasis have identified neurologic side effects likely due to occult cysticercosis. [5, 6] A detailed management plan for neurologic side effects from occult neurocysticercosis should be at the core of every mass drug administration program with praziquantel and/or ABZ in areas endemic for *T*. *solium*.

We wholeheartedly agree with the authors’ statement in their discussion that control programs applying chemotherapy using mass drug administration “need to be informed by evidence of the best drug and dose in terms of efficacy and side-effects.” Unfortunately, the flawed analysis that was published is contrary to that goal. Rather than promoting questionable conclusions based on weak evidence and inappropriate analyses, we believe that all stakeholders would be better served by pursuing more robust evidence to inform safe and effective interventions.
